# A Case of the Initial Presentation of Hemophagocytic Lymphohistiocytosis as Acute Unilateral Vision Loss

**DOI:** 10.7759/cureus.73820

**Published:** 2024-11-16

**Authors:** Georgia L Schafer, Yasaman Jaladat, Eman Hawy

**Affiliations:** 1 Ophthalmology, Loma Linda University Medical Center, Loma Linda, USA; 2 Pathology, Loma Linda University Medical Center, Loma Linda, USA; 3 Ophthalmology/Neuro-Ophthalmology, Loma Linda University Medical Center, Loma Linda, USA

**Keywords:** hemophagocytic lympho histiocytosis (hlh), neuro-opthalmology, ophthalmology case report, optic nerve infiltration, optic neuritis

## Abstract

Hemophagocytic lymphohistiocytosis (HLH) is an incredibly rare disease that is characterized by the overproduction of histiocytes and lymphocytes, which then start to attack the body instead of just invasive pathogens. It has been known to affect many organs including most commonly blood cells and bone marrow, but it has also been known to affect the liver, spleen, skin, and the brain. Ocular involvement is rare and is mostly associated with HLH’s effect on other body systems (i.e., HLH causes extremely low platelets which can lead to retinal hemorrhages). However, we discuss a case report of a 19-year-old man whose initial presentation of HLH was complete vision loss in one eye. MRI showed inflammation of the nerve which initially appeared similar to optic neuritis. Only after the patient began having systemic symptoms, as well as further lab tests, bone marrow biopsy, and imaging, were we able to make the diagnosis of HLH. This is the first reported case in the literature of a patient with HLH’s initial presenting symptom being acute unilateral vision loss. This finding is important for the ophthalmic community to be aware of, both because HLH needs to be included in the differential for rare causes of unilateral complete vision loss especially when associated with systemic atypical symptoms such as fever or altered mental status, and because early recognition of HLH by ophthalmologists could lead to life-saving treatment. If undiagnosed and untreated, HLH can be fatal in months.

## Introduction

Hemophagocytic lymphohistiocytosis (HLH) is a severe disease characterized by the overproduction of histiocytes and lymphocytes, which attack tissue or organs. It can be familial (genetic) or acquired secondary to an infection, malignancy, or autoimmune condition. The genetic form typically affects infants, while the secondary form is more common in the pediatric population and adults [1]. The incidence of primary HLH is estimated to be between one in 1 million births and one in 50,000 births [2,3]. HLH has started to be diagnosed far more frequently, which is likely due to increased recognition of the disease rather than an actual rise in incidence [4]. The acquired form of HLH is most commonly associated with Epstein-Barr virus (EBV) and has been associated with up to one-third of cases in studies from the United States [5]. It is a highly lethal disease, with studies showing that secondary HLH can have as high as a 76% mortality rate for high-risk patients within two months of their initial diagnosis [4]. 

HLH is a complex disease that can be very challenging to diagnose, often masquerading as other diseases. According to the HLH-2004 guidelines, five of the following eight criteria are needed to make a diagnosis of HLH: fever, splenomegaly, cytopenia, hypertriglyceridemia or hypofibrinogenemia, hemophagocytosis, low or absent NK-cell activity, hyperferritinemia, and high soluble IL-2 receptor levels [6].

HLH is a multi-system disease affecting many body organs, commonly bone marrow and blood cells. It can also affect the liver, skin, spleen, brain, and, rarely, the eyes. Wang et al. performed a large retrospective study of 1.5k patients with HLH. Of these patients, 341 had ophthalmic exams and 133 were found to have ocular abnormalities [7]. The most common ocular findings were retinal and vitreous hemorrhages. Other findings included many ocular pathologies which were a result of the systemic disease, rather than a result of a direct infiltration/attack of histiocytes to the ocular tissue [7]. We discuss the first reported case in the literature of a patient with HLH’s initial presenting symptom being acute unilateral vision loss secondary.

## Case presentation

Our patient is a 19-year-old man with a history of EBV who presented to an outside hospital for sudden vision loss in his right eye. MRI revealed right optic nerve enhancement. Given the severity of vision loss and the imaging findings, he was diagnosed with neuromyelitis optica (NMO), and he was treated with five days of 1 gram of Solumedrol/day and five days of plasmapheresis. No visual improvement was noted. Then, he received one gram of rituximab and was discharged. NMO and myelin oligoglycoprotein (MOG) antibody studies were negative. 

After he was discharged, he started to develop headaches, fevers, right-sided paresthesia, altered mental status (AMS), and difficulty ambulating. The patient was found to have pancytopenia. He was transferred to the Loma Linda University Medical Center for a higher level of care and Ophthalmology was consulted to assist with NMO management. 

On our initial evaluation, he had altered mental status and was A&Ox1 with a Glasgow Coma Scale (GCS) of 14 and was poorly cooperative due to extreme headache and light sensitivity. The vision was no light perception (NLP) OD (oculus dexter), 20/25 OS (oculus sinister). A large afferent pupillary defect (APD) was noted OD. Intra-ocular pressure was 11 OD and 24 OS. Confrontation visual fields were unable to be assessed OD and full OS. Color plates were 0/12 OD and 12/12 OS. The right optic nerve was mildly cupped at 0.4 c/d ratio with trace pallor, the left was at 0.2 c/d ratio with good color. The rest of the fundus exam was unremarkable.

**Figure 1:**
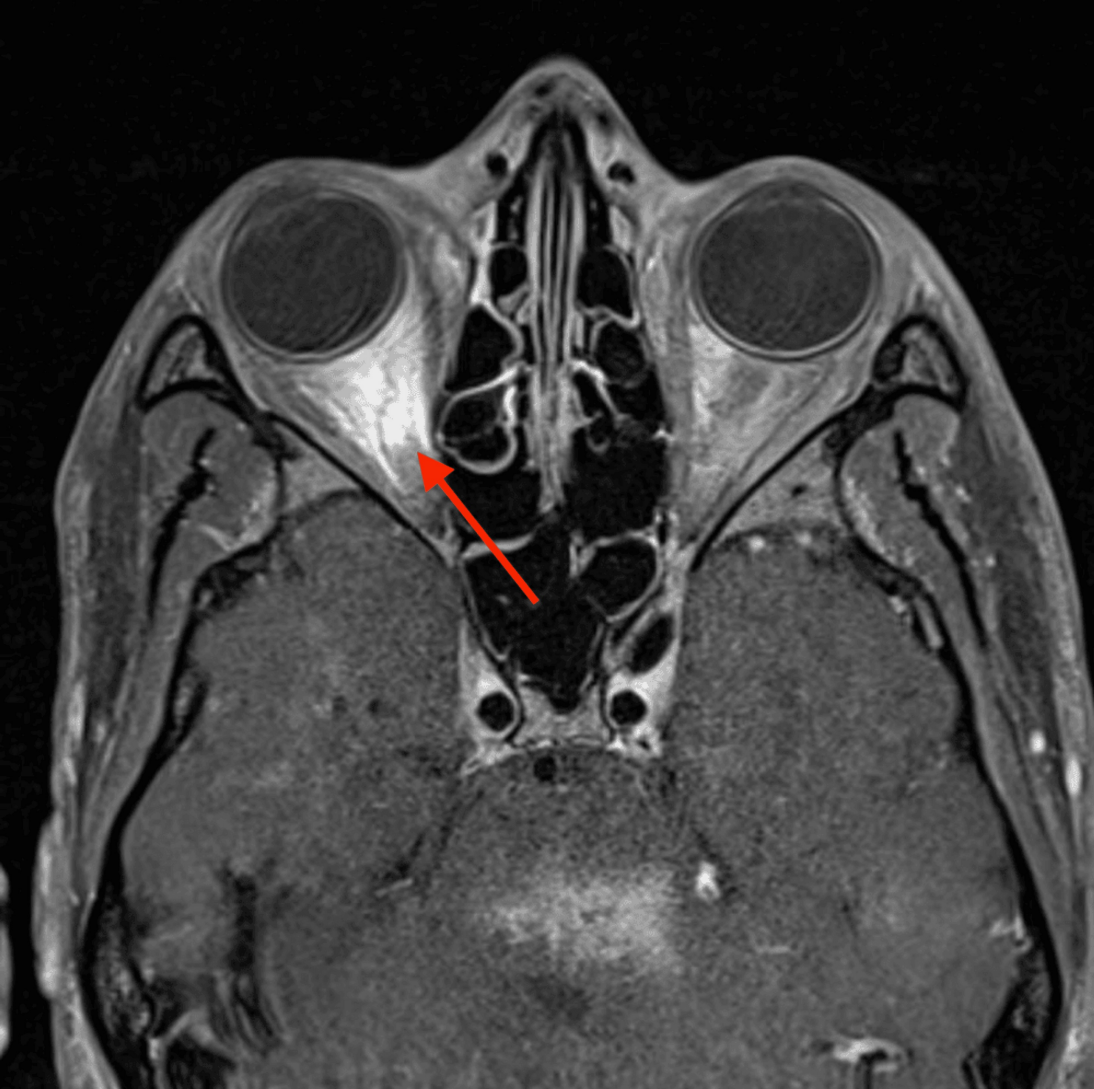
MRI orbits, T1 post contrast axial view. Right optic nerve and orbital fat enhancement.

MRI brain and orbit (Figure 1) showed orbital fat and optic nerve enhancement on the right side, multiple sites of hemorrhagic and tumefactive lesions in the brain in a spectral pattern consistent with HLH, demyelinating disease, or autoimmune/viral encephalitides. The hemorrhagic brainstem lesions were inconsistent with a typical presentation of multiple sclerosis (MS), myelin oligodendrocyte glycoprotein (MOG), or NMO.

Our patient had multiple systemic symptoms and lab findings that were not consistent with NMO. He also had no vision improvement after steroids, plasmapheresis, and rituximab. Our neuro-ophthalmology team recommended repeat NMO and MOG testing, which came back negative. Due to the low WBC and the presence of hemorrhagic brain lesions, as well as fever and AMS, rheumatology and hematology services were consulted for further workup of vasculitis or hematologic malignancies.

**Figure 2:**
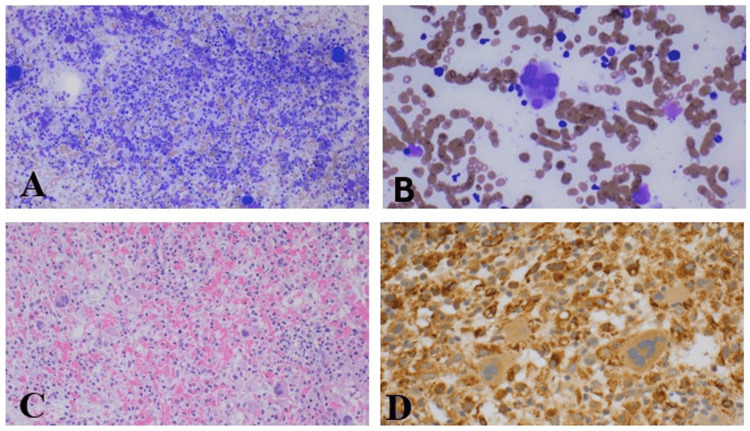
Bone marrow biopsy pathology. (A) Low-power photomicroscopy of bone marrow aspirate with scattered histiocytes with frequent hemophagocytosis. (B) High-power photomicroscopy of bone marrow aspirate with scattered histiocytes with frequent hemophagocytosis. (C) Low-power photomicroscopy of core biopsy showing a hypercellular marrow including scattered histiocytes with frequent hemophagocytosis. (D) The immunohistochemistry staining for CD 68 is positive.

Vasculitis workup was unrevealing. A bone marrow biopsy and lumbar puncture (LP) were performed. Bone marrow studies showed hypercellular marrow, frequent histiocytes, and scattered hemophagocytosis (Figure 2). There was no increase in blasts or marrow involvement concerning leukemia or lymphoma. Cerebral spinal fluid (CSF) studies were grossly normal. 

Notable lab tests included elevated triglycerides, IL-6, IL-18, soluble IL-2, ferritin, and low NK levels, as well as positive HLH genetic testing. Due to the patient meeting six of the eight criteria mentioned above, a diagnosis of HLH was made.

He was given three doses of IVIG and five days of 1 g/day of IV Solumedrol. Then he was started on HLH treatment which included etoposide IV 150 mg/m2 on days 1 and 4, Decadron PO 8mg BID on days 2-6, and intrathecal methotrexate. 

Three weeks after admission, the patient developed new-onset central vision loss in his left eye and was found to have a foveal central preretinal hemorrhage. His platelets at this time were 22k (normal range 150-450k). 

The patient was seen in the neuro-ophthalmology clinic after being discharged. Outpatient testing revealed significant right optic atrophy on optical coherence tomography (OCT) retinal nerve fiber layer (RNFL) (cnt 39 OD, cnt 109 OS) and continued NLP vision OD. Humfrey visual field testing revealed generalized depression OD and normal visual field OS. The preretinal hemorrhage was improved in the left eye.

## Discussion

HLH has been found to have ophthalmic complications in the past. However, it rarely presents with optic nerve infiltration. According to the findings from Wang et al's study, many ocular findings were not a result of a direct infiltration/attack of histiocytes or cytokines to the ocular tissue itself, but rather a result of the systemic disease [7]. For example, HLH commonly leads to extremely low platelet numbers which then can cause spontaneous retinal or vitreous hemorrhages. Another example is the severely depleted lymphocytes can allow for cytomegalovirus (CMV) flares leading to CMV retinitis.​ 

​The presentation of HLH in our patient’s left eye, with vision loss secondary to a retinal hemorrhage has been previously documented. However, the optic nerve findings that originally seemed to mimic an optic neuritis picture are far less common. Only a few case studies describe similar pathology. Shekarchian et al. described bilateral optic nerve infiltration in a nine-year-old with a known history of HLH which resulted in NLP OD and CF (counting fingers) OS vision with optic disc edema OU (oculus uterque) [8]. CSF studies revealed tumoral cells. His vision did not resolve with chemotherapy [8]. Chong et al. also detail the case of optic nerve infiltration seen on MRI in an eight-month-old with previously diagnosed familial HLH [9]. Vizcano et al. describe autopsy findings for three adult cases of HLH with vision impairment [10]. They found bilateral histiocytes with scant lymphocytes infiltrating the uvea with focal extension to the retina, optic nerve, and angle [10]. Other case reports have detailed various other ocular pathologies, but the optic nerve infiltration seems to be exceedingly rare. Our case seems to detail the first case of optic nerve infiltration as the primary presenting symptom of HLH.​

According to the HLH 2004 therapeutic guidelines, treatment should include dexamethasone, chemotherapy including etoposide and cyclosporine A, and in patients with concern for CNS involvement intrathecal methotrexate and corticosteroids [6]. Studies have not investigated the effect of intrathecal chemotherapy on long-term vision prognosis in cases with optic nerve involvement.​

## Conclusions

This leads to the important question, do ophthalmologists need to include HLH in a differential for complete unilateral vision loss in patients who are exhibiting unusual systemic symptoms? The answer is yes, not because this is a common diagnosis, but because it can be quickly fatal within months if missed. Correctly diagnosing ocular HLH will help future patients obtain treatment in a more timely fashion.

Since this is a rare diagnosis that is starting to be recognized more often, there are still many gaps in the literature, especially with regard to HLH's effects on the eyes. Future studies would be beneficial to evaluate the efficacy of HLH treatment on final visual symptoms in patients with optic nerve involvement if treatment is started in a timely fashion. It would also be helpful to look at the prevalence of optic nerve involvement with HLH patients in a retrospective case series.

## References

[1] (2024). Hemophagocytic lymphohistiocytosis (HLH). ed.

[2] Meeths M, Horne A, Sabel M, Bryceson YT, Henter JI (2015). Incidence and clinical presentation of primary hemophagocytic lymphohistiocytosis in Sweden. Pediatr Blood Cancer.

[3] Ishii E, Ohga S, Imashuku S (2007). Nationwide survey of hemophagocytic lymphohistiocytosis in Japan. Int J Hematol.

[4] Abdelhay A, Mahmoud AA, Al Ali O, Hashem A, Orakzai A, Jamshed S (2023). Epidemiology, characteristics, and outcomes of adult haemophagocytic lymphohistiocytosis in the USA, 2006-19: a national, retrospective cohort study. EClinicalMedicine.

[5] Marsh RA (2017). Epstein-Barr virus and hemophagocytic lymphohistiocytosis. Front Immunol.

[6] Henter JI, Horne A, Aricó M (2007). HLH-2004: diagnostic and therapeutic guidelines for hemophagocytic lymphohistiocytosis. Pediatr Blood Cancer.

[7] Wang L, Suo L, Kou F (2023). Ocular phenotypes in patients with hemophagocytic lymphohistiocytosis: a retrospective analysis in a single center over 7 years. Am J Ophthalmol.

[8] Shekarchian F, Abadi MK, Shariati MM (2023). Clinical approach to a child with hemophagocytic lymphohistiocytosis and bilateral optic nerve head infiltration: a case report and brief literature review. Clin Case Rep.

[9] Chong KW, Lee JH, Choong CT, Paeds MM, Chan DW, Fortier MV, Chan MY (2012). Hemophagocytic lymphohistiocytosis with isolated central nervous system reactivation and optic nerve involvement. J Child Neurol.

[10] Vizcaino MA, Eberhart CG, Rodriguez FJ (2017). Hemophagocytic lymphohistiocytosis in adults with intraocular involvement: clinicopathologic features of 3 cases. Ocul Oncol Pathol.

